# Histology of the Pharyngeal Constrictor Muscle in 22q11.2 Deletion Syndrome and Non-Syndromic Children with Velopharyngeal Insufficiency

**DOI:** 10.1371/journal.pone.0021672

**Published:** 2011-06-28

**Authors:** Josine C. C. Widdershoven, Nicole E. Spruijt, Wim G. M. Spliet, Corstiaan C. Breugem, Moshe Kon, Aebele B. Mink van der Molen

**Affiliations:** 1 Department of Otolaryngology, Head and Neck Surgery, Maastricht University Medical Center, Maastricht, The Netherlands; 2 Department of Plastic Surgery, University Medical Center Utrecht, Utrecht, The Netherlands; 3 Department of Pathology, University Medical Center Utrecht, Utrecht, The Netherlands; Harvard Medical School, United States of America

## Abstract

Plastic surgeons aim to correct velopharyngeal insufficiency manifest by hypernasal speech with a velopharyngoplasty. The functional outcome has been reported to be worse in patients with 22q11.2 deletion syndrome than in patients without the syndrome. A possible explanation is the hypotonia that is often present as part of the syndrome. To confirm a myogenic component of the etiology of velopharyngeal insufficiency in children with 22q11.2 deletion syndrome, specimens of the pharyngeal constrictor muscle were taken from children with and without the syndrome. Histologic properties were compared between the groups. Specimens from the two groups did not differ regarding the presence of increased perimysial or endomysial space, fiber grouping by size or type, internalized nuclei, the percentage type I fibers, or the diameters of type I and type II fibers. In conclusion, a myogenic component of the etiology of velopharyngeal insufficiency in children with 22q11.2 deletion syndrome could not be confirmed.

## Introduction

The 22q11.2 deletion syndrome (22q11.2DS) is the most common human microdeletion syndrome [1] with an estimated frequency around 1 in 4000 [2] but possibly as high as 1 in 2000 surviving newborns [3]. It encompasses the phenotypes previously known as DiGeorge syndrome, velocardiofacial syndrome, conotruncal anomaly face syndrome, many cases of the autosomal dominant Opitz G/BBB syndrome, and Cayler cardiofacial syndrome (asymmetric crying facies). Over 180 clinical features including every organ system have been associated with the deletion [4].

One of the presenting features of 22q11DS is velopharyngeal insufficiency (VPI). Velopharyngeal insufficiency is the failure of the soft palate to reach the posterior pharyngeal wall to close the opening between the oral and nasal cavities, resulting in hypernasal speech. Incomplete velopharyngeal closure is most frequently related to structural abnormalities such as cleft palate or submucous cleft, but may also be the corollary of neuromuscular impairment [5]. Both seem to be factors in the etiology of VPI in patients with 22q11.2 deletion syndrome where palatal defects, adenoid hypoplasia, and platybasia enlarge the pharyngeal gap [6], and the hypodynamic pharynx as viewed by nasendoscopy has been described as a “black hole” [7].

Surgical repair of palatal clefts does not sufficiently correct VPI in 10- 31.8% of all patients with VPI not restricted to those with 22q11DS [8], [9], [10], [11], [12], possibly due to stiffness or shrinkage of the velum due to scarring [5]. Secondary velopharyngoplasty to correct the VPI may then follow. The functional outcome has been reported to be worse in patients with 22q11DS than in patients without the syndrome [13], [14], [15], [16], [17], [18]. A possible explanation is the hypotonia that is often present as part of the syndrome and which cannot be corrected by surgery.

Velopharyngeal closure is achieved by the concert action of multiple muscles, including palatal lift by the levator veli palatini and circular pharyngeal closure by the pharyngeal constrictor muscle (PCM) [19], [20]. A previous study of the PCM, shows that patients with 22q11DS have proportionally more type I fibers and the diameter of these fibers is smaller than those in people without the syndrome [21]. In the study by Zim et al, muscle biopsies from children were compared with specimens from elderly cadavers.

Muscle fiber hypoplasia or atrophy with subsequent pharynx hypotonia may be primarily myogenic or neurogenic. Muscular and neurologic problems have been associated with 22q11DS both clinically and genetically. Specific myopathies are rare [22], [23], [24], but neurologic disorders including delayed motor and mental development [25], [26], [27] and dysfunction of cranial nerves III, VII, VIII, IX, X, and XII [28] affect at least 33% of patients [29], [30]. General hypotonia, which affects 23-76% of patients with 22q11DS [29], [31], [32], was found to be universally prevalent among children with 22q11DS and VPI [33].

About 40 genes [3], including TBX1, are located in the 3.0 megabase region deleted in 22q11DS [1], affecting countless downstream signaling pathways. The central roles of the TBX1 and CRKL genes in the anomalous developmental of pharyngeal structures in 22q11DS have recently been reviewed [34]. The murine Tbx1−/− model for 22q11DS has hypoplastic branchiomeric muscles [35], [36], but the sporadic muscles that develop have a normal distribution of muscle fibers types [37]. In patients with 22q11DS, decreased PCM muscle thickness on MRI [21] suggests hypoplasia. The temporal Tbx1 gradient follows the cranial-caudal development of pharyngeal structures [36], causing structures that are derived from more cranially located pharyngeal arches, such as the levator palatini muscles, to be less affected by the mutation than structures derived from more caudally located pharyngeal arches, such as the PCM muscle [38], [39]. Although Tbx1 is not expressed in primary neural crest cells [40], Tbx1 mutants have aberrant structures derived from neural crest cells including cranial nerves [38] since defective Tbx1 expression in the pharyngeal endoderm affects the downstream expression Fgf8 and Fgf10 which are necessary for neural crest cell migration [38], [41], [42]. As suggested by studies on the deleted TBX1 gene [35], [37], [38], primary aberrant myogenesis leads to aberrant neurogenesis.

In summary, the poorer functional outcome after velopharyngoplasty in patients with 22q11DS may be attributed to pharyngeal hypotonia. Anomalous myogenesis and neurogenesis which may underlie the hypotonia have been reported in a murine model for 22q11DS. In this study we aimed to confirm a myogenic component of the etiology of VPI in children with 22q11DS by analyzing the histology of the PCM muscle. Our clinical experience is that the PCM seems thicker in children with 22q11DS. We expect to find fiber hypertrophy as a corollary of the muscle hypoplasia [35], [36] necessitating the few fibers present to take on a heavier workload.

## Methods

### Ethics Statement

This study was approved by the institutional medical ethics review board (Utrecht University Medical Center Ethics Review Board) and the patients' parents gave written informed consent to participate.

### Patients

The University Medical Centre in Utrecht is the Dutch national centre for children with 22q11DS. Children undergoing velopharyngoplasty for VPI with and without the 22q11DS were included in the study. Children with contra-indications for velopharyngoplasty (including bleeding disorders or extensive comorbidity such as cardiac problems) and known neurological disorders were excluded.

### Sample size calculation

Using the results of the only previous study on PCM histology in 22q11DS [21] which found a difference of mean diameter of type I fibers of 5.0 µm between patients with and without 22q11DS, with a standard deviation of 2.0 µm, an alpha of 0.05, and a power of 0.80 in the two-tailed two-sample t-test sample size formula yields a sample size of 4 subjects in each group. This number was arbitrarily doubled as the difference between two groups of children is likely smaller than the difference between children and adults in the previous study.

### Muscle specimens

During velopharyngoplasty, a cranially attached pharyngeal flap (measuring around 10×40−50 mm) is mobilized from the dorsal pharyngeal wall and attached to the velum. This flap is comprised of part of the PCM muscle and the overlying mucosa. Muscle at the caudal end of the flap is trimmed (measuring around 10×3 mm) and delivered fresh to the pathologist in a damp gauze for histological evaluation.

### Outcome parameters

Histological evaluation of the muscle specimens included qualitative analysis and quantitative measurements. The analysts were blinded for age, gender and presence of the syndrome. The specimens were qualitatively evaluated for the presence of increased perimysial and endomysial space, muscle fiber grouping by size or type, and presence of internalized nuclei. After staining with ATPase at pH 4.3, representative areas from each specimen were photographed. For quantitative analysis, muscle fibers were counted and the percentage of type I muscle fiber was calculated per patient. The diameters of up to 100 fibers of each type were measured for each patient. For each muscle fiber type, the mean fiber diameter and variance ((SD×1000)/mean diameter) were calculated per group (males, females, and children with and without 22q11DS).

### Statistical analysis

The genders of children with and without 22q11DS were compared using the Chi-square test. Age at surgery of males and females and children with and without 22q11DS were compared using the Independent samples t-test. The presence of increased perimysial and endomysial space, muscle fiber grouping by size and type, and internalized nuclei was compared between the two groups using Fisher's exact test. The relationship between age at surgery and fiber diameters was examined using the Spearman correlation. The independent samples t-test was used to compare the mean percentage of type I fibers and muscle fiber diameters between males and females and between children with and without 22q11DS.

## Results

### Patients

**Figure 1:**
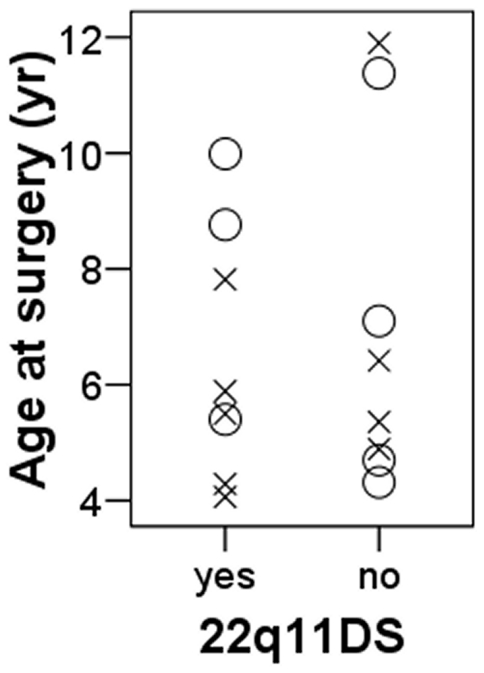
Group demographics. O: males, X: females.

Muscle specimens were available for 16 children, eight with 22q11DS and eight without 22q11DS. The groups did not differ regarding gender (5/8 = 63% and 4/8 = 50% female, respectively, p = 0.63) or age at surgery (6.5 and 7.0 years, respectively, p = 0.68) (Figure 1). Males and females did not differ regarding age at surgery (7.4 and 6.2 years, respectively, p = 0.39).

### Qualitative analysis

**Figure 2:**
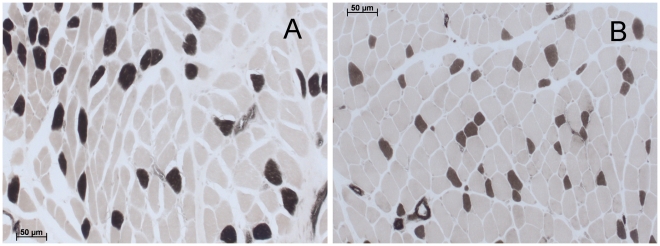
Histological specimens with ATPase stain at pH 4.3. A, a 5-year-old female without 22q11DS but with increased perimysial and endomysial space. B, a 10-year-old male with 22q11DS and without increased perimysial and endomysial space. Bars 50 µm.

No structural differences were seen between histological specimens from children with and without 22q11DS (Table 1, Figure 2). Increased perimysial and endomysial space was seen equally in both groups. No grouping by muscle fiber type was seen in any patient. One non-syndromic patient had localized grouping of smaller fibers, but these were round fibers without nuclear clumping which do not suggest neurogenic atrophy or other signs of fiber degeneration and regeneration. One patient with 22q11DS had an increased percentage of internalized nuclei.

**Table 1:** Qualitative analyses.

Parameter	22q11DS(n = 8)	No 22q11DS(n = 8)	p
Increased perimysial space, No. (%)	5 (63)	5 (63)	1
Increased endomysial space, No. (%)	4 (50)	6 (75)	0.61
Grouping by size, No. (%)	0 (0)	1 (13)	1
Grouping by fiber type, No. (%)	0 (0)	0 (0)	1
Internalized nuclei, No. (%)	1 (13)	0 (0)	1

### Quantitative measurements

**Figure 3:**
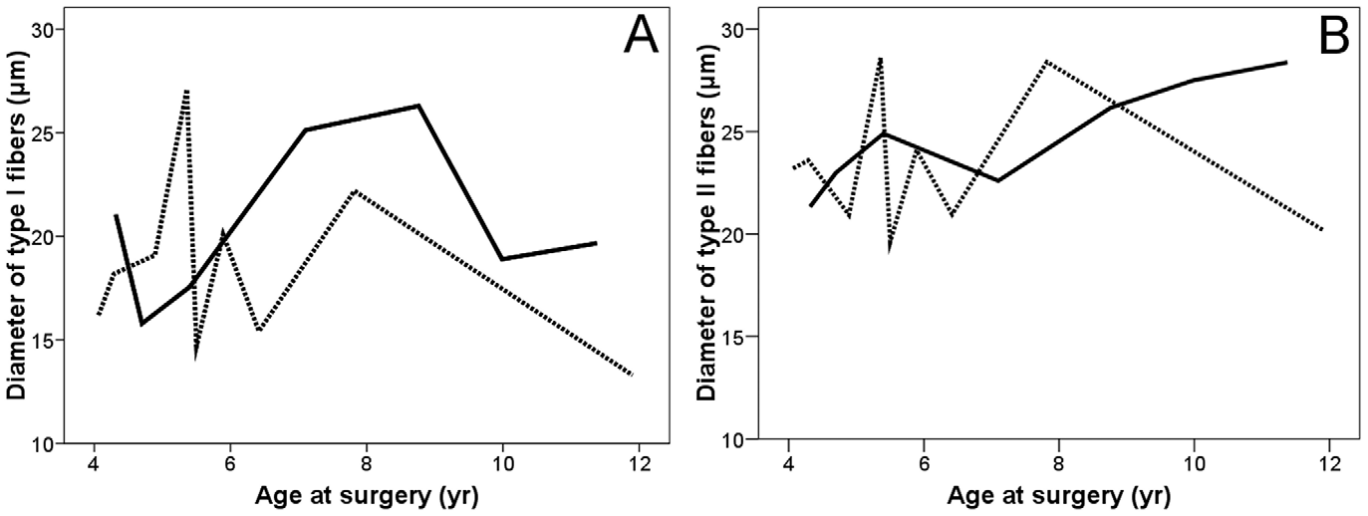
Mean diameters of type I (A) and type II (B) muscle fibers and age at surgery. Solid lines: males, dashed lines: females.

**Figure 4:**
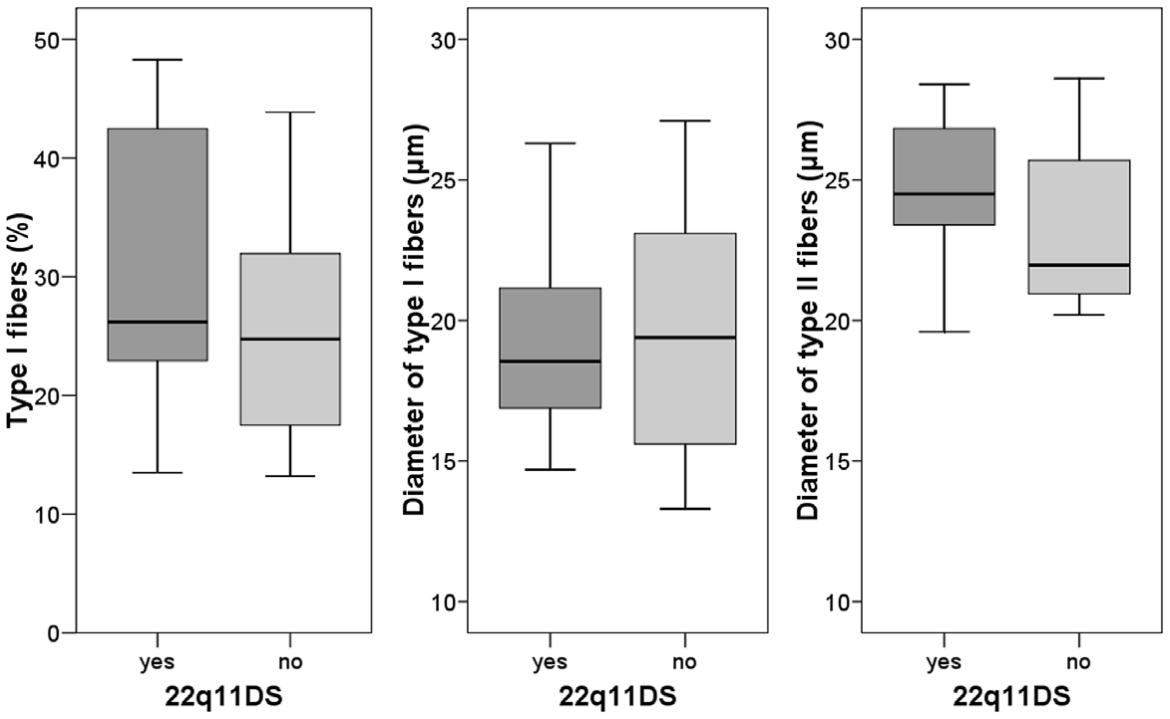
Muscle fiber type measurements for children with and without 22q11DS. Bands, means. Boxes, 25^th^–75^th^ percentiles. Whiskers, 95% confidence intervals.

There was no correlation between muscle fiber diameter and age at surgery (p = 0.78 for type I fibers and p = 0.48 for type II fibers, Figure 3). Neither the percentage of type I fibers nor the diameters of the fiber types differed significantly between males and females or between children with and without 22q11DS (Table 2, Figure 4). All calculated fiber diameter variances were less than 250 (Table 2). For all groups, the mean diameters of type I fibers were more than 12% smaller than the mean diameters of the larger type II fibers.

**Table 2:** Quantitative analyses.

Parameter	Male	Female	Mean difference (95% CI)	p	22q11DS	No 22q11DS	Mean difference (95% CI)	p
Type I fibers, % (SD)	24.8 (10.3)	30.7 (11.9)	−6 (−18, 6)	0.43	30.6 (12.3)	25.7 (10.5)	4.9 (−7, 17)	0.46
Type I fiber diameter, µm (SD),	20.6 (3.9),	18.5 (4.3),	2 (−2, 7)	0.32	19.3 (3.7),	19.6 (4.8),	−0.3 (−5, 4)	0.92
variance	189	232			192	245		
Type II fiber diameter, µm (SD),	24.8 (2.6),	23.3 (3.3),	2 (−2, 5)	0.37	24.7 (2.8),	23.3 (3.4),	1.4 (−2, 5)	0.25
variance	105	142			113	146		

## Discussion

Few studies have looked at the histology of the PCM. With the exception of specimens obtained from patients undergoing pharyngoplasty [21] or laryngectomy [43], most only study specimens from cadavers.

### Morphology

Our qualitative analysis revealed no morphologic differences between PCM muscles in children with and without 22q11DS (Table 1). We found increased perimysial and endomysial space in both groups. While increased space is associated with chronic muscle damage, it is unclear whether this is also true for pharyngeal constrictors. Since it affects both groups equally, it is unlikely to be a factor in the poorer speech in children with 22qDS. Zim et al. [21] found increased endomysial space in children with 22q11DS relative to adults without the syndrome, but did not test the difference for significance. Like Zim et al. [21], we did not find any grouping by muscle fiber type, indicating the absence of innervation distubances.

### Fiber type

We found 30.6% (SD 12.3) and 25.7% (SD 10.5) type I muscle fibers, respectively, in children with and without 22q11DS. Zim et al. [21] found 27.7% (SD 2.01) and 17.9% (SD 2.15) type I muscle fibers, respectively, in children with and adults without 22q11DS. The significant difference between the groups in the study by Zim et al. may not necessarily be attributed to the presence of the syndrome, but may be distorted by the unusually small percentage of type I fibers found in the adult controls (81–86 years, cadavers). Other studies on pharyngeal constrictor specimens in adults found 35% (43–77 years, live) [43], 49% (SD 9.2) (38–61 years, cadavers) [44], and 33.7% (SD 12.0) (over 50 years, cadavers) [45] type I fibers. Leese and Hopwood [45] report 20.4% (SD 8.7) type I fibers in infants (0–3 years) and 30.2% (SD 15.3) type I fibers in young adults (12–49 years). While they report no significant change with respect to age, they also report that infant muscle fibers exhibit a significantly lesser percentage of type I fibers.

### Fiber diameter

Previous reports on the mean diameter of type I muscle fibers in pharyngeal constrictor muscles in adults without 22q11DS range from 26.6 to 29 µm [21], [44]. In children without 22q11DS we found a mean diameter of 19.6 µm (SD 4.8). In children with 22q11DS, Zim et al. [21] found a mean diameter of 21.6 µm (SD 2.09) and we found a mean diameter of 19.3 µm (SD 3.7). It is tempting to conclude that, as with limb muscles, mean fiber diameter is related to age [46]. However, we did not find a correlation between age and diameter among children of different ages (Figure 3) and Leese and Hopwood [45] failed to find a relationship among adults of different ages. They did find a significant difference between fiber diameters in infants (0–3 years) and adults (over 12 years). Like Leese and Hopwood [45], we found no difference in fiber diameter between males and females, reflecting similar usage of the muscles by both genders.

The similar diameters of both type I and II muscle fibers in children with and without 22q11DS found in this study reflect similar strain put on this muscle by all children with VPI. Unfortunately, we did not have a control group of PCM specimens from children without VPI. Presumably, children without structural abnormalities that lead to VPI will have smaller muscle fiber diameters as they have do not have to employ the pharyngeal muscles as vigorously to close the oropharynx off from the nasopharynx.

Fiber type disproportion, reflected in a difference between the mean fiber type diameters of more than 12% of the mean diameter of the larger fiber type, is characteristic of congenital myopathies [46]. In this study, the type II fibers were more than 12% larger than the type I fibers in both children with and without 22q11DS. In the study by Zim et al. [21], the diameters of the type II fibers were also more than 12% larger than the type I fibers in children with 22q11DS, while the muscle fiber types had similar diameters in adults without 22q11DS. The disproportion is likely a result of selective type II hypertrophy rather than type I atrophy as children with VPI place extra strain on the fast type II fibers while attempting to articulate properly and preventing nasal regurgitation while swallowing.

We found greater variance in muscle fiber diameter (192 and 113) than Zim et al. [21] (97 and 77, respectively, for type I and II fibers in children with 22q11DS). Our measurements are based on more fibers per patient (171 to 200) than the study by Zim et al. [21] (64 to 113 fibers per patient). We found greater variance among children without 22q11DS (245 and 146, respectively, for type I and II fibers), but no groups had variances greater than 250, which is considered pathologic in limb muscles, but has been found in healthy palatal muscles [47].

### Conclusion

Therefore, we conclude that there is no evidence of innervation or myogenic disturbances in the histologic specimens of the PCM in children with 22q11DS relative to non-syndromic counterparts. The absence of histologic deficits in the PCM muscle of patients with 22q11DS does not preclude the functional deficits manifest in the hypodynamic pharynx seen on nasendoscopy and poorer functional outcome after velopharyngoplasty. Future studies to elucidate the etiology of the pharyngeal hypotonia in 22q11DS should investigate the role of the central nervous system, such as by comparing fMRI images taken during speech. Meanwhile, unanswered etiologic and clinical questions hamper adequate management of the compromised speech understandability in patients with 22q11DS, contributing to poor social functioning and quality of life.
